# Account of Diseases in an Eastern District of London, from the 20th of March to the 20th of April, 1801

**Published:** 1801-05

**Authors:** 


*Account of Diseases in an Eastern District of London, from the 20th of March to the 20th of April,* 1801.

| ACUTE DISEASES. | No. of Cases. | CHRONIC DISEASES. | No. of Cases. |
|---|---|---|---|
| Typhus - - - - - - | 25 | Cough - - - - - - - | 23 |
| Pneumonia - - - - - - | 3 | Dyspnœa - - - - - | 12 |
| Febris Hectica - - - - | 1 | Cough and Dyspnœa - | 13 |
| Ophthalmia - - - - - | 2 | Catarrhus Senilis - - - | 2 |
| Apoplexy - - - - - - | 1 | Phthisis Pulmonalis - - | 10 |

| | |
|---|---|
| Pertuſſis | 1 |
| Hydrothorax | 2 |
| Aſcites | 1 |
| Anaſarca | 3 |
| Cephalæa | 6 |
| Hemicrania | 1 |
| Vertigo | 6 |
| Paralyſis | 1 |
| Hepatitis Chronica | 3 |
| Diarrhœa | 20 |
| Dyſenteria | 3 |
| Enterodynia | 3 |
| Gaſtrodynia | 2 |
| Nephritis | 1 |
| Dyſuria | 2 |
| Fluor Albus | 6 |
| Amenorrhœa | 4 |
| Chloroſis | 5 |
| Aſthenia | 3 |
| Lumbago | 1 |
| Sciatica | 2 |
| Cynanche Parotidœa | 3 |
| Rheumatiſmus Odontalgicus | 4 |
| Rheumatiſmus Chronicus | 10 |
| PUERPERAL DISEASES. | |
| Menorrhagia Lochialis | 4 |
| Lactea | 2 |
| Dolores Poſt Partum | 3 |
| Abſceſſus Mammæ | 1 |
| INFANTILE DISEASES. | |
| Convulſio | 2 |
| Vermes | 3 |
| Febris Infantilis | 1 |
| Diarrhœa | 4 |
| Pertuſſis | 1 |

The diſeaſe which has ſo long engaged the attention of the medical practitioner, and which has been conſidered as the reigning epidemic, ſtill continues in a conſiderable degree to prevail. The number of patients affected by this fever has within the laſt few weeks, rather increaſed than diminiſhed. Affections of the head, which have been repeatedly referred to, as a common ſymptom in typhus, but as prevailing in a peculiar degree in the late fever, ſtill continues as a prominent feature of the diſeaſe. Pain in the head, coma, or delirium, have been protracted longer than uſual, and have even continued after other febrile ſymptoms have abated. Complaints of the head have indeed lately occurred in a great number of caſes, as an idiopathic diſeaſe. Violent pains, confuſion of ideas, and vertigo, have been frequently complained of, where no ſymptoms of fever could be diſcovered; and vertiginous affections have in ſeveral inſtances approached very nearly to an apoplexy.

The north-eaſterly winds which blew a few weeks ago, were productive of different complaints in the face and jaws. Slight Ophthalmias alſo have been experienced by perſons who had been for a conſiderable time expoſed to their influence. The diſeaſe denominated by noſologiſts Cynanche Parotidœa, and vulgarly called the Mumps, has alſo been frequently troubleſome, and may probably be attributed to a ſimilar cauſe. Rheumatic pains of the face and teeth, termed Rheumatiſmus Odontalgicus, have alſo occurred in ſeveral inſtances.

MONTHLY

